# Hot Corrosion of NiCrAlY and NiCrAlY/YSZ Coatings Under Na_2_SO_4_ and Na_2_SO_4_ + NaCl Salt Deposits at 900 °C

**DOI:** 10.3390/ma19091701

**Published:** 2026-04-23

**Authors:** Youbei Sun, Jianjiang Zhao, Xiufang Gong, Bin Long, Yubing Pei, Wei Wang, Juanqiang Ding, Hua Wei

**Affiliations:** 1 Polytechnic Institute, Zhejiang University, Hangzhou 310027, China; richonf@163.com; 2 Dongfang Electric Corporation Dongfang Turbine Co., Ltd., Deyang 618000, China; gongxiufang@dongfang.com (X.G.); longbin@dongfang.com (B.L.); peiyubing@dongfang.com (Y.P.); wangweidq@dongfang.com (W.W.); dingd8586@163.com (J.D.); 3 State Key Laboratory of Clean and Efficient Turbomachinery Power Equipment, Deyang 618000, China; 4 Institute of Hypergravity Science and Technology, Zhejiang University, Hangzhou 310058, China; zjjiang@zju.edu.cn

**Keywords:** hot corrosion, NiCrAlY coatings, thermal barrier coating, sulfate–chloride attack, nickel-based superalloys

## Abstract

**Highlights:**

**What are the main findings?**

- Bare M247 alloy suffers rapid catastrophic hot corrosion under Na_2_SO_4_ and Na_2_SO_4_ + NaCl, dominated by basic fluxing and active oxidation mechanisms, respectively.
- NiCrAlY coating forms a dense α-Al_2_O_3_ protective scale, endowing M247 alloy with excellent hot corrosion resistance.
- NiCrAlY/YSZ coating exhibits the optimal corrosion resistance due to the physical and thermal barrier effects of the YSZ layer.

**What are the implications of the main findings?**

- Clarify the anti-corrosion mechanisms of two coatings, providing an experimental basis for the structural design of high-temperature protective coatings.
- Verify that high Al content is the key to MCrAlY coatings forming stable protective oxide scales in sulfate–chloride environments.
- Offer technical guidance for improving the service life of high-temperature components like gas turbine blades in corrosive atmospheres.

**Abstract:**

Two types of coatings, NiCrAlY and NiCrAlY/YSZ, were fabricated on the surface of M247 alloy by the atmospheric plasma spraying (APS) technique. Under pure Na_2_SO_4_ and 25 wt.% NaCl-containing mixed salt deposits at 900 °C in air, the M247 alloy underwent rapid catastrophic corrosion. The non-protective corrosion products formed on the surface included NiO and (Ni,Co)Cr_2_O_4_ spinel. The hot corrosion of M247 under the pure Na_2_SO_4_ salt deposit followed a basic fluxing mechanism, whereas under the NaCl-containing mixed salt deposit, it was dominated by an active oxidation mechanism. During hot corrosion, the NiCrAlY coating developed a continuous, dense, and highly protective α-Al_2_O_3_ oxide scale on its surface, endowing it with superior hot corrosion resistance. The thermal barrier coating of NiCrAlY/YSZ exhibited the best hot corrosion resistance, attributed to the physical barrier and thermal barrier effects of the outer YSZ ceramic layer.

## 1. Introduction

The degradation of nickel-based superalloys under high-temperature corrosive environments remains a critical challenge for gas turbine engines operating in marine or industrial atmospheres, where sulfate and chloride deposits accelerate material failure via hot corrosion mechanisms [1,2,3]. Mar-M247, a γ′-strengthened Ni-based superalloy, is widely utilized in turbine blades due to its exceptional mechanical strength at elevated temperatures [4]. However, prolonged exposure to Na_2_SO_4_ and NaCl-containing environments at temperatures above 900 °C induces catastrophic corrosion via fluxing reactions, leading to rapid depletion of protective oxide scales and subsurface sulfidation [5,6,7]. To mitigate this, protective coatings such as MCrAlY (M = Ni, Co) overlays and thermal barrier coating (TBC) systems incorporating yttria-stabilized zirconia (YSZ) have been extensively studied [8,9,10]. NiCrAlY coatings provide oxidation resistance through the formation of Al_2_O_3_ scales, while YSZ-based TBCs reduce surface temperatures and inhibit salt penetration [11,12,13]. Nonetheless, the synergistic effects of mixed sulfate–chloride deposits and cyclic thermal stresses at extreme temperatures (≥1000 °C) often lead to premature coating spallation and accelerated substrate attack [14,15,16].

Recent studies highlight the critical role of coating architecture in resisting Type I (800–950 °C) and Type II (600–800 °C) hot corrosion [17,18,19]. NiCrAlY bond coats demonstrate superior resistance to Na_2_SO_4_-induced corrosion compared to aluminide coatings due to their ability to regenerate protective oxides under cyclic conditions [20]. However, chloride infiltration through coating defects can destabilize the Al_2_O_3_ scales via volatile metal chloride formation, accelerating corrosion kinetics [21,22,23]. The addition of YSZ as a topcoat introduces a thermal gradient and physical barrier against salt deposition, yet phase instability (e.g., tetragonal-to-monoclinic transformation) and CMAS (CaO-MgO-Al_2_O_3_-SiO_2_) infiltration at elevated 1000 °C remain persistent issues [24,25,26]. Furthermore, the interaction between Na_2_SO_4_ + NaCl melts and YSZ at elevated temperatures may accelerate zirconia destabilization, creating pathways for corrosive species to reach the bond coat [27,28].

While numerous studies have evaluated MCrAlY coatings or YSZ-based TBCs individually [29], systematic comparisons of monolithic NiCrAlY versus bilayer NiCrAlY/YSZ coatings in mixed sulfate–chloride environments above 900 °C are scarce. Most existing works either focus on single salt systems or lack direct performance benchmarking between single-layer and bilayer coating architectures under identical corrosive conditions. Adding NaCl to Na_2_SO_4_ lowers its melting point. Zhang et al. [30] further quantified the effect of adding 25 wt.% NaCl on the melting point of the salt film and investigated the thermal corrosion behavior of cobalt-based alloys and NiCrAlYSi coatings in this composite salt environment. Gond et al. [31] demonstrated that YSZ coatings delay salt penetration but are susceptible to cracking under thermal cycling. However, the temperature-dependent transition from oxidation-dominated degradation to sulfidation–chlorination mechanisms remains poorly understood for these coating systems. Moreover, the corrosion mechanisms of M247 superalloy under the combined action of Na_2_SO_4_ and NaCl at 900 °C have not been systematically distinguished, leading to ambiguity in targeted coating design. This study fills a research gap by investigating the thermal corrosion behavior of NiCrAlY and NiCrAlY/YSZ coatings on Mar-M247 alloy surfaces under Na_2_SO_4_ and Na_2_SO_4_ + NaCl salt film environments at 900 °C. It provides the first direct comparative data on corrosion resistance in pure sulfate and sulfate–chloride composite salt systems, elucidating the enhancement mechanism of the YSZ overlay in complex corrosion environments. Furthermore, it systematically distinguishes the corrosion resistance of M247 high-temperature alloy in pure Na_2_SO_4_ and Na_2_SO_4_ + NaCl environments, demonstrating the corrosion resistance of the NiCrAlY overlay in composite corrosion conditions–chloride composite salt systems, elucidating the enhancement mechanism of the YSZ top layer in composite corrosion environments. Furthermore, it systematically distinguishes the corrosion mechanisms of M247 high-temperature alloy under pure Na_2_SO_4_ and Na_2_SO_4_ + NaCl composite salt conditions, providing a theoretical basis for designing corrosion-resistant coatings for turbine components. Advanced characterization techniques, including scanning electron microscopy (SEM), energy dispersive spectroscopy (EDS) and X-ray diffraction (XRD), elucidate the corrosion evolution and mechanisms, providing insights for coating design in next-generation turbines.

## 2. Materials and Methods

### 2.1. Fabrication of NiCrAlY and NiCrAlY/YSZ Coating

Based on prior research on thermal barrier coatings (TBCs), a NiCrAlY bond coat was selected due to its dual role in protecting the substrate from oxidation and mitigating thermal expansion mismatch between the ceramic top coat and the M247 superalloy substrate. The ceramic layer consisted of yttria-stabilized zirconia, a widely adopted material for TBCs applications. Ingots of the M247 alloy with a nominal composition of Ni-0.15C-10Co -8.25Cr-0.7Mo-10W-5.5Al-1Ti-0.5Fe1.5Hf-1.5Ta-0.05Zr-0.015B (wt.%) were wire-cut into cylindrical specimens with a Φ18 × 2 mm. Prior to coating, substrates were grit-blasted with alumina (Al_2_O_3_) to enhance adhesion and ultrasonically cleaned in ethanol to remove surface contaminants. The bonding layer (NiCrAlY) and top ceramic layer (YSZ) were prepared using atmospheric plasma spraying (APS) and high-velocity oxygen fuel (HVOF) techniques. Spraying parameters were as follows: spraying distance 130–140 mm, voltage 104–106 V, current 450–460 amperes, Ar/H_2_ plasma gas flow 45/5 L/min, carrier gas flow 2.5–2.8 L/min, powder feed rate 30–40 g/min. In this study, the NiCrAlY metal binder layer had the composition Ni 32 wt.%, Cr 21 wt.%, Al 8 wt.%, Y 0.5 wt.%, Co balance, with a powder particle size of 15–45 µm. The powder composition for the YSZ ceramic coating was 8 wt.% yttrium oxide + 72 wt.% zirconium oxide, with a particle size of 53–106 µm.

### 2.2. Hot Corrosion Testing

Hot corrosion experiments were conducted in a horizontal tube furnace under static air. Two corrosive media were employed: (1) pure Na_2_SO_4_ salt and (2) a mixed salt of 75 wt.% Na_2_SO_4_ + 25 wt.% NaCl. Both salts were pre-dried at 120 °C for 24 h, ground into fine powders, and uniformly dispersed (3 mg/cm^2^) onto the surfaces of three sample types: bare M247 alloy, NiCrAlY-coated M247, and TBC systems (NiCrAlY/YSZ). Samples were placed in alumina crucibles and loaded into the furnace. To ensure constant weight measurement, crucibles were pre-fired at 900 °C for 10 h until mass stabilization. Corrosion tests were carried out at 900 °C for 100 h. During the experiment, the sample was weighed every 4 h for the first 20 h, followed by intervals of 10 h and then 20 h. After removal, it was allowed to cool at room temperature for 2 h. After confirming that the sample temperature had fully returned to room temperature, the total mass change was measured using an analytical balance with an accuracy of 0.0001 g. Na_2_SO_4_ was purchased from Tianjin Benchmark Chemical Co., Ltd., and NaCl was obtained from Sinopharm Group Co., Ltd. All reagents were used as received without further purification.

### 2.3. Microstructural and Chemical Characterization

To preserve oxide scale integrity during sample preparation, corroded samples were electroplated with a thin Ni layer (~3 μm) via electroless deposition prior to cold-mounting in epoxy resin. Mounted specimens were mechanically ground to 2000-grit SiC paper and polished with 0.05 μm colloidal silica. Phase identification of as-sprayed and corroded samples was performed using a Philips powder diffractometer (Rigaku D/Max 2500v, Japan) CuKα, λ=1.54178Å over a 2θ range of 20–80° at a scanning rate of 5°/min. Cross-sectional and surface microstructures were analyzed via scanning electron microscopy SEM, JSM−7800F operated in backscattered electron (BSE) mode. Elemental distributions and localized chemistry were probed using energy dispersive X-ray spectroscopy (EDX, Oxford Instruments, UK) with an Oxford detector.

## 3. Results

### 3.1. Characteristic of NiCrAlY and NiCrAlY/YSZ Coating

**Figure 1:**
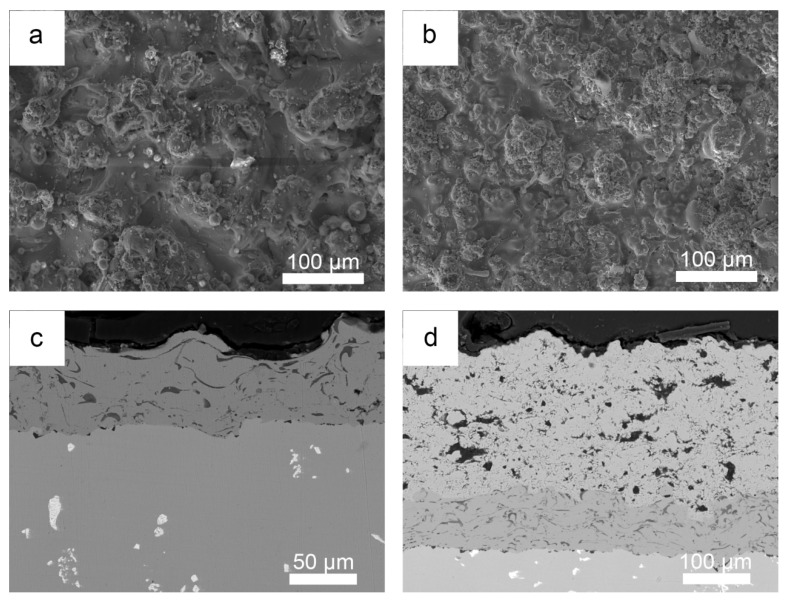
Surface and cross-sectional morphologies of the APS plating NiCrAlY (**a**,**c**) and NiCrAlY/YSZ (**b**,**d**) coatings on the M247 substrate.

**Figure 2:**
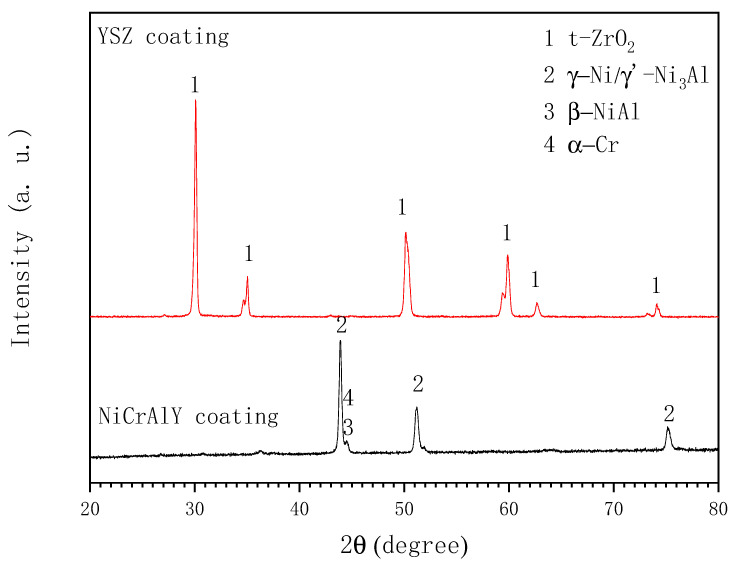
X-ray diffraction patterns of the two coatings.

Figure 1 shows surface and cross-sectional morphologies of the APS plating NiCrAlY (Figure 1a,c) and NiCrAlY/YSZ (Figure 1b,d) coatings on the M247 substrate. The surfaces of the two coatings show that both coatings are composed of particles from a few microns to tens of microns in size, and no obvious holes and cracks are found. This is due to the APS technology that uses a 5–50 micron alloy/ceramic powder to form the coatings on the surface of the substrate under high temperature and high speed. The cross-sectional coatings indicate that the NiCrAlY bond coating is comparatively dense, about 70 µm in thickness and uniformly structured with no cracks, and it has good adhesion to the substrate. However, the ceramic YSZ coating displays a distinctive cauliflower-like microstructure containing microcracks and porosity. This morphological configuration can enhance thermal insulation capabilities through its inherent defect architecture, while simultaneously improving thermal shock resistance via stress accommodation mechanisms enabled by these structural discontinuities. The coexistence of such microstructural features demonstrates an optimized balance between thermal barrier functionality and mechanical durability in service conditions. The ceramic layer is about 280 µm in thickness, and it also remains strongly adhesive to the MCrAlY coating. EDX analysis show that the NiCrAlY coating has an average composition of 61.2 Ni, 25.9 Cr, 11.3 Al, 1.6 Y (wt.%), and the XRD pattern in Figure 2 identifies that phase composition of the bond coating mainly includes Ni and Ni3Al, and a small quantity of -NiAl, and -Cr phases. The ceramic layer exhibits distinct diffraction peaks attributable to a single tetragonal ZrO_2_ phase without an obvious monoclinic ZrO_2_ phase, confirming effective t-ZrO_2_ phase stabilization through yttria doping. The absence of monoclinic zirconia peaks in the diffraction patterns suggests complete phase transformation during the deposition process, which is critical for maintaining the coating’s thermo-mechanical stability. This phase configuration aligns with the characteristic requirements for high-performance thermal barrier coatings in corrosive environments. The mass ratio of Zr to Y in the ceramic layer is about 14.4:1 by EDX analysis (not shown here), which reveals that the content of Y_2_O_3_ in the ceramic layer is 6 wt.%.

### 3.2. Hot Corrosion Under Pure Na_2_SO_4_ Salt Deposit

**Figure 3:**
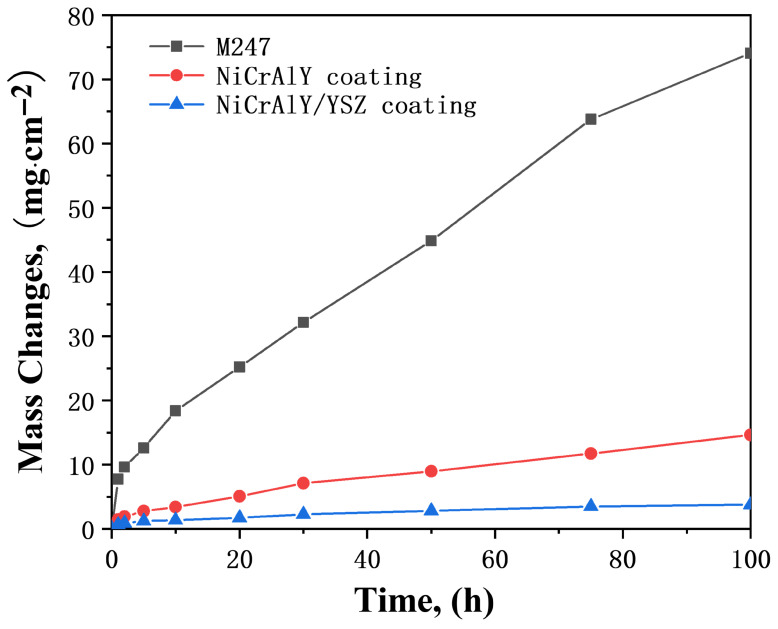
Corrosion kinetics of the M247, the NiCrAlY coating and the NiCrAlY/YSZ coating under 3 mg/cm^2^ Na_2_SO_4_ salt deposit in air at 900 °C.

**Figure 4:**
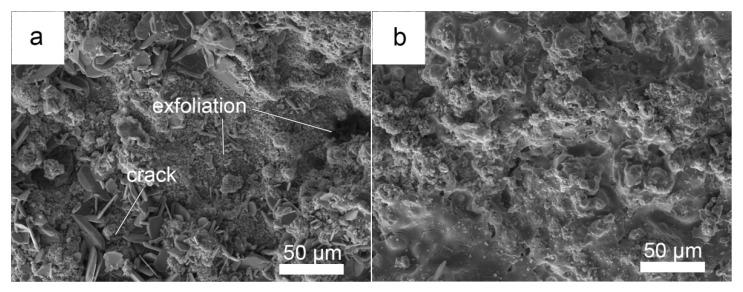
Surface morphologies of the NiCrAlY coating (**a**) and the NiCrAlY/YSZ coating (**b**) under 3 mg/cm^2^ Na_2_SO_4_ salt deposit after corroded in air at 900 °C for 100 h.

**Figure 5:**
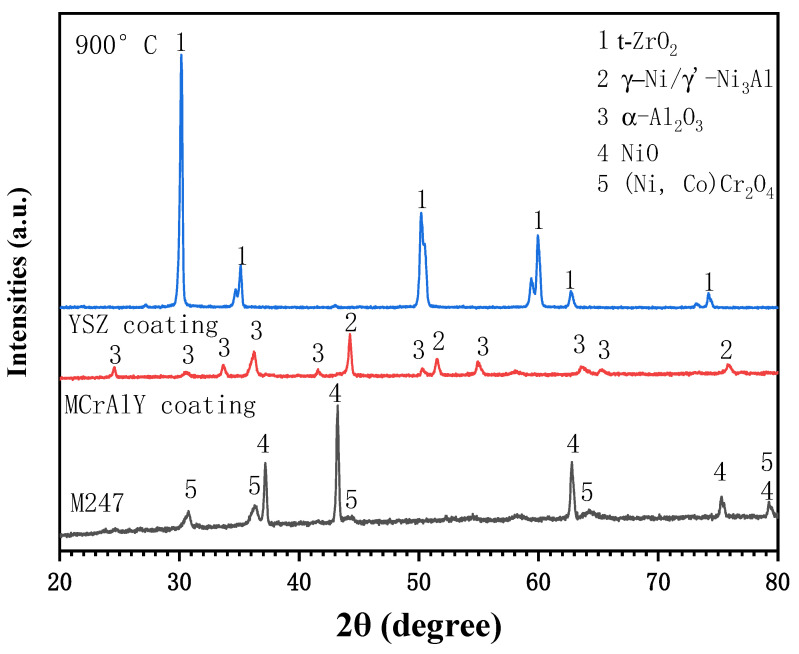
XRD patterns of the M247, the NiCrAlY coating and the NiCrAlY/YSZ coating under 3 mg/cm^2^ Na_2_SO_4_ salt deposit after corroded in air at 900 °C for 100 h.

**Figure 6:**
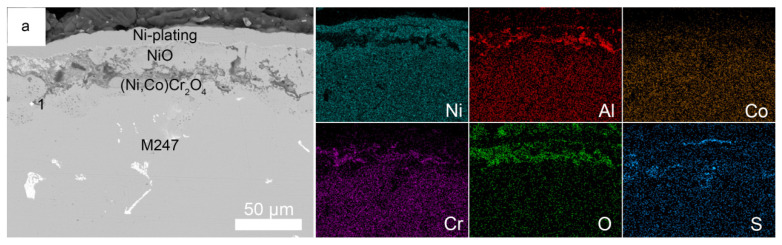

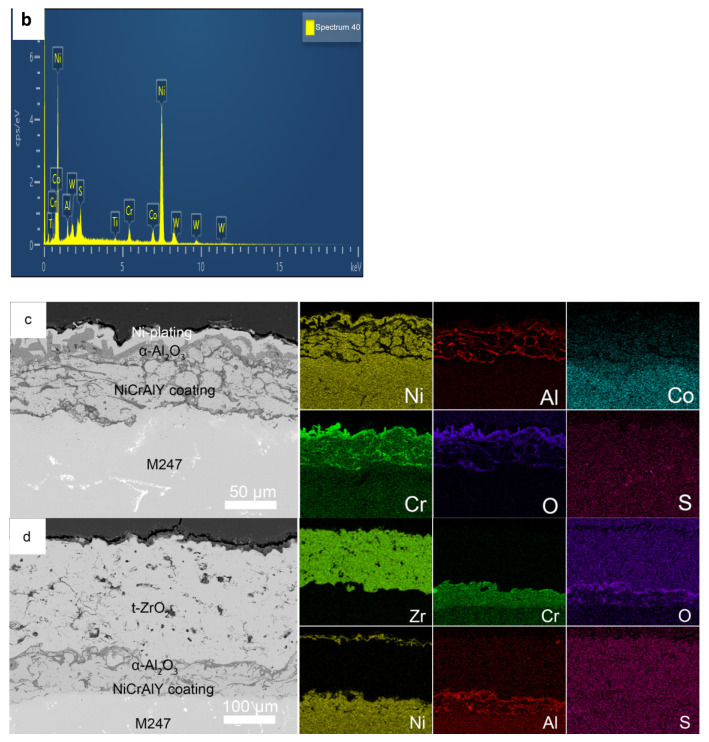
Cross-sectional morphologies of the M247 (**a**), the NiCrAlY coating (**c**) and the NiCrAlY/YSZ coating (**d**) under 3 mg/cm^2^ Na_2_SO_4_ salt deposit after corroded in air at 900 °C for 100 h; (**b**) EDS analysis at point 1 marked in the M247 alloy cross-section.

Corrosion kinetics of the M247, NiCrAlY coating, and NiCrAlY/YSZ coating with 3 mg/cm^2^ Na_2_SO_4_ deposit in air at 900 °C are shown in Figure 3. After undergoing rapid corrosion during the initial 2 h of hot corrosion, the corrosion rate of the M247 base alloy slowed down. By the end of the 100 h hot corrosion process, the corrosion weight gain reached approximately 74.0 mg/cm^2^, and no incubation period was observed at the beginning of the hot corrosion. During the hot corrosion process, severe cracking, pulverization, and spalling of corrosion products occurred on the surface of the M247 alloy specimen, so the surface morphology photograph of the corroded M247 alloy is not presented. In contrast, the morphologies of the corroded NiCrAlY coating and NiCrAlY/YSZ coating are shown in Figure 4. Analysis by surface XRD (Figure 5) and cross-sectional photographs (Figure 6a) revealed that the small amount of corrosion products consisted of two layers. The outer bright layer was primarily composed of NiO, while the discontinuous dark inner layer was mainly (Ni, Co)Cr_2_O_4_. A continuous protective Cr_2_O_3_ or Al_2_O_3_ scales could not be formed because the Cr and Al contents in M247 are only 8.25 and 5.5 wt.%, respectively, which are far lower than the contents required to form protective Cr_2_O_3_ or Al_2_O_3_ oxide scales (at least 15 and 10 wt.%, respectively) [3,5]. Consequently, M247 experienced rapid corrosion under the Na_2_SO_4_ deposit, indicating poor resistance to sulfate-induced hot corrosion. products on the specimen surface were observed. Only a small amount of corrosion products remained on the surface after the corrosion process, as shown in the cross-sectional photograph in Figure 6. Notably, a small amount of S enrichment was found near the corrosion product/matrix alloy interface (Figure 6b). EDX analysis revealed that this enrichment mainly consisted of NiS. No obvious internal oxidation was observed in the base alloy after 100 h of hot corrosion.

However, the hot corrosion kinetic curves of both the NiCrAlY coating and the thermal barrier coatings (NiCrAlY/YSZ coating) are close to parabolic. After 100 h, the corrosion weight gain of the former is close to 14.6 mg/cm^2^, while that of the latter is 3.7 mg/cm^2^. Through fitting, the parabolic rate constant kp of the former is approximately 5.5 × 10^−10^g^2^⋅cm^−4^⋅s^−1^, and that of the latter is 3.7 × 10^−11^ g^2^⋅cm^−4^⋅s^−1^. The former rate constant is one order of magnitude higher than the latter, indicating that the NiCrAlY monolayer coating exhibits relatively faster corrosion kinetics compared to the NiCrAlY/YSZ dual-layer coating. For the surface morphology of the NiCrAlY coating after hot corrosion (Figure 4a), slight cracking and spalling of surface corrosion products can be observed. Surface XRD (Figure 5) and cross-sectional images (Figure 6b) show that the corrosion products on the surface of the NiCrAlY coating mainly consist of a continuous, dense, and well-adherent Al_2_O_3_ scale. This Al_2_O_3_ oxide scale provides good protection against hot corrosion for the coating. No interdiffusion is found between the coating and the substrate alloy, and the bonding between coating and substrate is excellent.

After 100 h of hot corrosion, the surface of the thermal barrier coating (NiCrAlY/YSZ coating) (Figure 4b) shows no obvious changes or spalling compared with that before corrosion (Figure 1b). Surface XRD (Figure 5) and cross-sectional images (Figure 6c) indicate that no corrosion products are detected on the ceramic layer surface, and only a continuous, dense, and well-adherent Al_2_O_3_ scale (thermal gravity oxides, TGO) is formed between the ceramic layer (YSZ coating) and the bond coating (NiCrAlY coating). The bonding between the ceramic layer and the TGO, the TGO and the bonding layer, and the bonding layer and the substrate alloy is good, with no obvious interdiffusion observed. This suggests that the TBC (NiCrAlY/YSZ coating) exhibits the best resistance to Na_2_SO_4-_induced hot corrosion, which is attributed to the physical barrier and thermal barrier effects of the ceramic layer against corrosive species, and this will be discussed in detail when analyzing the protective mechanism of the coatings later. It is worth noting that the surface XRD results reveal that the ceramic layer remains a single tetragonal phase t-ZrO_2_ after 100 h of hot corrosion, without transforming into the monoclinic phase m-ZrO_2_, indicating the excellent high-temperature stability of the ceramic layer in Na_2_SO_4_ hot corrosion processes.

### 3.3. Hot Corrosion Under a Mixed Salt Deposit of 75 wt.% Na_2_SO_4_ + 25 wt.% NaCl

**Figure 7:**
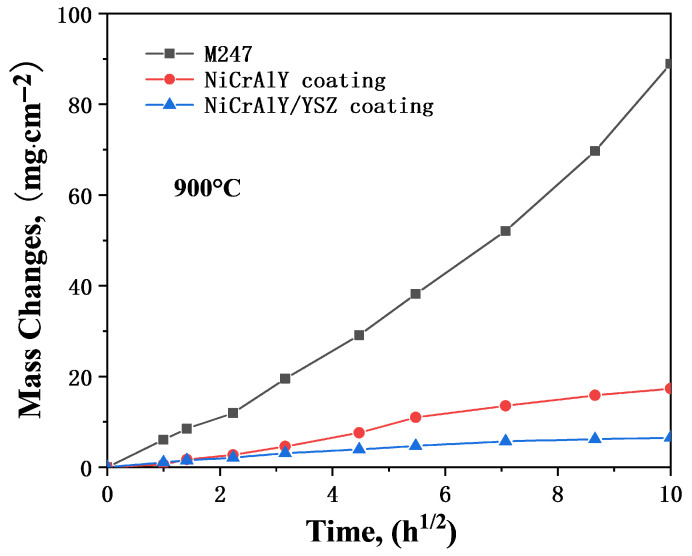
Corrosion kinetics of the M247, the NiCrAlY coating and the NiCrAlY/YSZ coating under 3 mg/cm^2^ Na_2_SO_4_ + 25 wt.% NaCl salt deposit in air at 900 °C.

Figure 7 presents the corrosion kinetics curves of the M247 alloy, NiCrAlY coating, and NiCrAlY/YSZ coating under a 3 mg/cm^2^ of 75 wt.% Na_2_SO_4_ + 25 wt.% NaCl mixed salt deposit at 900 °C in air. Similar to the corrosion kinetics under pure Na_2_SO_4_ deposit, the M247 alloy undergoes rapid corrosion within the initial 2 h, followed by a slowdown corrosion procession. In contrast, corrosion kinetics of both the NiCrAlY coating and the NiCrAlY/YSZ coating exhibit obvious parabolic laws. However, the hot corrosion of M247, NiCrAlY coating, and NiCrAlY/YSZ coating under the mixed salt deposit is more severe than that under pure Na_2_SO_4_ salt deposit. The corrosion weight gains after 100 h of hot corrosion reach 88.9, 17.3, and 6.5 mg/cm^2^, respectively, showing significant increases. The parabolic rate constants fitted from the corrosion kinetics of the NiCrAlY coating and NiCrAlY/YSZ coating are 1.0 × 10^−9^ and 1.2 × 10^−10^ g^2^⋅cm^−4^⋅s^−1^, respectively, both of which are higher than those under the Na_2_SO_4_ salt deposit.

**Figure 8:**
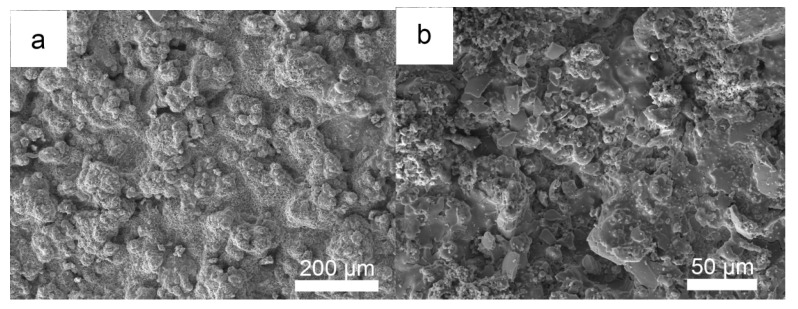
Surface morphologies of the NiCrAlY coating (**a**) and the NiCrAlY/YSZ coating (**b**) under 3 mg/cm^2^ Na_2_SO_4_ + 25 wt.% NaCl salt deposit after corroded in air at 900 °C for 100 h.

**Figure 9:**
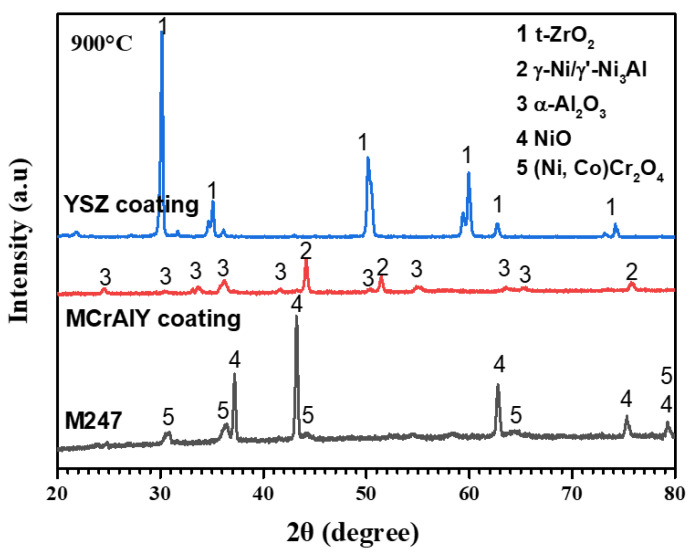
XRD patterns of the M247, the NiCrAlY coating and the NiCrAlY/YSZ coating under 3 mg/cm^2^ Na_2_SO_4_ + 25 wt.% NaCl salt deposit after corroded in air at 900 °C for 100 h.

**Figure 10:**
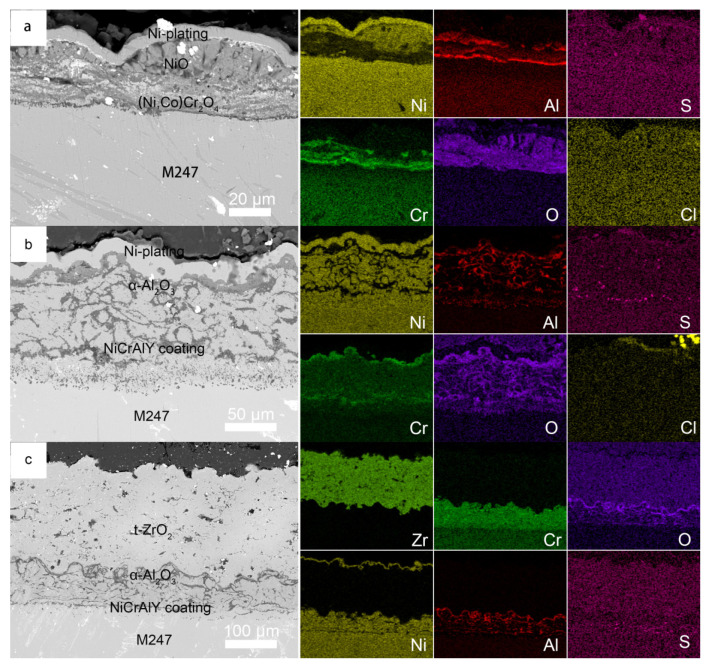
Cross-sectional morphologies of the M247 (**a**), the NiCrAlY coating (**b**) and the NiCrAlY/YSZ coating (**c**) under 3 mg/cm^2^ Na_2_SO_4_ + 25 wt.% NaCl salt deposit after corroded in air at 900 °C for 100 h.

After 100 h of hot corrosion under the mixed salt, the surface photograph of the NiCrAlY coating (Figure 8a) shows no obvious cracking or spalling. XRD characterization of its surface corrosion products (Figure 9) combined with cross-sectional morphology observation (Figure 10b) reveals that a continuous, dense, and well-adherent Al_2_O_3_ protective scale is formed on the surface, endowing the NiCrAlY coating with excellent hot corrosion resistance to the mixed salt. The surface morphology (Figure 8b), XRD characterization (Figure 9), and cross-sectional morphology (Figure 10c) of the NiCrAlY/YSZ coating after hot corrosion show that no corrosion products are found on the ceramic layer surface, and only a continuous, dense, and well-adherent Al_2_O_3_ protective scale is formed at the interface between the ceramic layer and the bond coating. This reveals that the thermal barrier coating has the best hot corrosion resistance to the mixed salt. Moreover, the ceramic layer is single tetragonal t-ZrO_2_, also indicating that the ceramic layer has excellent high-temperature stability under the mixed salt deposit.

## 4. Discussion

In high-temperature environments, whether superalloys and coatings exhibit good oxidation/corrosion resistance depends on whether a protective Cr_2_O_3_, Al_2_O_3_, and SiO_2_ scale can form on their surfaces. Generally, both alloys and coatings contain oxidation/corrosion-resistant components such as Cr, Al and other elements. When the surfaces of alloys and coatings are covered by molten salts, each component therein will undergo very complex chemical reactions with oxygen in the molten salts and air at high temperatures, generating a corrosion layer including corresponding metal oxides, sulfides, and complex oxides. Depending on the composition of the alloys and coatings, as well as the composition of the molten salts, the corrosion layer may be a protective Cr_2_O_3_ or Al_2_O_3_ scale, or non-protective metal oxides (FeO, NiO, and Cr_2_O_3_), spinels (NiCr_2_O_4_, NiAl_2_O_4_), or metal sulfides (FeS, NiS, and Co_2_S_3_) [32].

In this study, the nickel-based superalloy M247 suffered catastrophic corrosion under pure Na_2_SO_4_ salt deposit due to insufficient Cr and Al contents. The corrosion products were mainly a mixture of NiO, (Ni, Co)Cr_2_O_4_, and NiS. These products were loose, prone to cracking and spalling, and non-protective, allowing the hot corrosion process to continue. Additionally, although the high contents of W, Ti, and Mo in M247 are beneficial for its high-temperature mechanical properties, they easily induce acidic corrosion during hot corrosion [3], which is another reason for the poor hot corrosion performance of M247. There are three main mechanisms for sulfate-induced hot corrosion: the sulfidation model [33], the acid-base fluxing model [34], and the electrochemical model [35]. After analyzing the results of this experiment, the corrosion mechanism of M247 can be explained by the acid-base fluxing model. This theory suggests that, at 900 °C, Na_2_SO_4_ (melting point: 884 °C) on the alloy surface is in a molten state. The corrosion process begins with the decomposition of Na_2_SO_4_:

Na_2_SO_4_ = Na_2_O + SO_3_ = Na_2_O + SO_2_ + 1/2O_2_ (1)

Due to the high oxygen partial pressure in the air, it is difficult for Reaction (1) to occur at the molten salt/air interface. However, at the alloy/molten salt interface, where the oxygen partial pressure is very low, Reaction (1) readily occurs, simultaneously generating the basic oxide Na_2_O. At this interface, the metallic components M=Ni, Co, Cr, Al, etc. in the alloy undergo oxidation reactions with the O_2_ generated in Reaction (1):

M + 1/2O_2_ = MO (2)

According to Le Chatelier’s principle, as oxygen is consumed, Reaction (1) shifts to the right, increasing the concentration of Na_2_O at the alloy/molten salt interface. This Na_2_O then reacts with the oxides formed in Reaction (2):

Na_2_O + MO = 2Na^+^ + MO_2_^2−^ (3)

The generated MO_2_^2−^ migrates from the alloy/molten salt interface to the molten salt/air interface. At the molten salt/air interface, due to the high oxygen partial pressure and low Na_2_O concentration, the migrated MO_2_^2−^ decomposes, precipitating loose MO (i.e., the corrosion layer):

MO_2_^2−^ = MO + O^2−^ (4)

**Figure 11:**
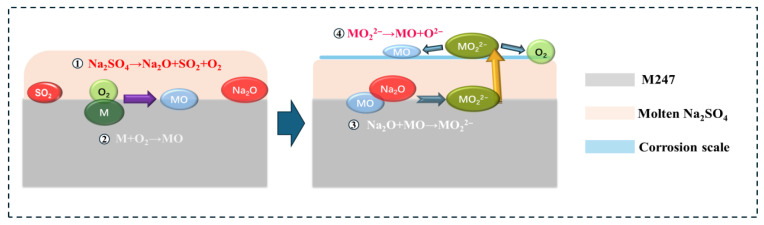
The basic fluxing process of Na_2_SO_4-_induced hot corrosion.

This is the basic fluxing process of hot corrosion (seen in Figure 11). As analyzed above, a negative gradient in O^2−^ activity (or Na_2_O concentration) from the alloy/molten salt interface to the molten salt/air interface is a necessary condition for the basic fluxing process. These reactions continue until the salt deposit on the alloy surface is exhausted, at which point the accelerated corrosion of the metal ceases. Therefore, the corrosion layers formed by hot corrosion are generally porous, prone to spalling, and usually non-protective. During the basic fluxing process, sulfidation reactions of alloy components also occur simultaneously:

M + SO_2_ = MS + O_2_ (5)

This explains why sulfur-containing compounds are often detected at the alloy/corrosion product interface in most sulfate-induced hot corrosion cases.

However, when the deposited salt on the M247 surface was changed from pure Na_2_SO_4_ to a mixed salt of 75 wt.% Na_2_SO_4_ + 25 wt.% NaCl, the hot corrosion kinetics became faster, accompanied by more severe cracking, pulverization, and spalling of the surface corrosion products. A protective Cr_2_O_3_ or Al_2_O_3_ oxide film still did not form on the surface, and no sulfides or chlorides were detected in the corrosion products. This indicates that NaCl is more aggressive to the alloy than Na_2_SO_4_ during hot corrosion. However, for NaCl, Na^+^ has negligible oxidizing potential, and Cl^−^ only has reducing potential, so neither Na^+^ nor Cl^−^ can directly oxidize metallic components in M247 (such as Ni, Co, Cr, and Al). Shinata and Nishi heated a mixture of Cr and NaCl powders in pure Ar at 300–1200 K; no corrosion products were detected by differential thermal analysis [36]. The above analysis and results suggest that NaCl accelerates the hot corrosion process indirectly through some mechanism. A widely accepted explanation is that, when a mixed salt deposit exists on the M247 surface, the molten mixed salt (with a melting point lower than that of pure Na_2_SO_4_ and the hot corrosion temperature) causes NaCl to react with metal components or metal oxides on the alloy surface and oxygen in the air through the following chemical reactions:

2NaCl + MO + 1/2O_2_ = Na_2_MO_2_ + Cl_2_ (6)

2NaCl + M + O_2_ = Na_2_MO_2_ + Cl_2_ (7)

The generated Cl_2_ penetrates the molten salt and reacts with metallic components in the alloy at the alloy/molten salt interface through chlorination reactions:

M + Cl_2_ = MCl (8)

The generated metal chloride MCl_2_ volatilizes at high temperatures and diffuses outward to the molten salt/air interface with higher oxygen partial pressure, where it is re-oxidized to form metal oxides and chlorine gas:

MCl_2_ + 1/2O_2_ = MO + Cl_2_ (9)

**Figure 12:**
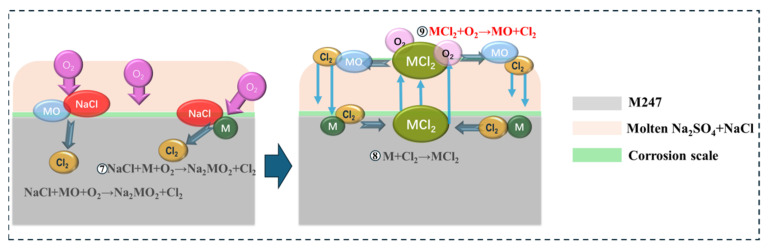
The “activation–oxidation” mechanism of chloride-induced hot corrosion.

The regenerated Cl_2_ can then penetrate back to the alloy/molten salt interface to react with metallic components, forming a self-sustaining accelerated hot corrosion process (seen in Figure 12). This is the “activation–oxidation” mechanism of chloride-induced hot corrosion [37].

Most metal chlorides have high volatility at high temperatures, and gas diffusion in molten salts is faster than ionic diffusion, so chloride-induced hot corrosion kinetically proceeds more rapidly, with greater corrosion weight gain and more easily spalling corrosion products than sulfate-induced hot corrosion [38]. Therefore, in this study, the activation–oxidation mechanism caused by NaCl was dominant in hot corrosion process induced by mixed salt. The absence of chlorides in the corrosion products can be attributed to two reasons: One is the volatilization of chlorides after the molten salt is exhausted, and the other is that any residual chlorides in the corrosion products may have been dissolved by water during cross-sectional sample preparation.

When an APS-prepared NiCrAlY coating was applied to the M247 surface, molten salt-induced acid-base fluxing process and activation–oxidation mechanisms occurred under Na_2_SO_4_ and mixed salt deposits, respectively. However, the high Al content (11 wt.%) in the coating, the third-element effect of Cr [39], and the reactive element effect of Y [40] promoted the selective oxidation of Al at the initial stage of hot corrosion, forming a uniform, dense, and well-adhered protective α-Al_2_O_3_ oxide scale. This α-Al_2_O_3_ scale is highly stable in molten salts, hinders the penetration of corrosive elements such as S, Cl, and O [41] and prevents further corrosion of metallic elements in the coating. Therefore, the NiCrAlY coating provided effective hot corrosion protection for the M247 alloy substrate. Lou et al. [42] found that, when sputtered CoCrAlY coatings with as low as 6 wt.% Al were hot-corroded in Na_2_SO_4_ molten salts with different NaCl contents, α-Al_2_O_3_ oxide films formed at the initial stage of corrosion, and increasing the Al content in the coating significantly improved its long-term resistance to NaCl-induced hot corrosion. Zhang et al. [30] studied the hot corrosion behavior of arc ion-plated NiCrAlYSi coatings under Na_2_SO_4_ and mixed salt deposits and found that a protective α-Al_2_O_3_ oxide film formed on the coating surface when the Al content was 8.6 wt.%, endowing it with good resistance to NaCl-containing mixed salt hot corrosion [43]. These studies indicate that, when the Al content in MCrAlY coatings exceeds 10 wt.%, a protective α-Al_2_O_3_ oxide scale can form under sulfate- and chloride-containing mixed salt deposits, thereby enhancing the coatings’ hot corrosion resistance.

When an APS-prepared TBC (NiCrAlY/YSZ) was applied to the M247 surface, its hot corrosion behavior under Na_2_SO_4_- and NaCl-containing mixed salts approached pure oxidation, with corrosion weight gain and kinetic rate constant (kp) values lower than those of the single NiCrAlY coating. This can be attributed to two main reasons: first, the physical barrier effect of the surface ceramic YSZ coating against corrosive media. In this study, the prepared ceramic coating was highly stable in both molten salts, and XRD analysis before and after hot corrosion showed a single tetragonal t-ZrO_2_ phase, and no corrosion products or phase transformation products were observed in surface or cross-sectional images. Although the top ceramic layer in TBC typically contains micro-voids and cracks to accommodate thermal expansion mismatch with the thermally grown oxide (TGO) and bond coating (NiCrAlY), which helps relieve stress during preparation and service and improve thermal shock resistance [44], these defects could potentially act as transport channels for corrosive media during hot corrosion. However, the ceramic layer thickness in this study reached 280 μm, making it difficult for the two molten salts to penetrate, and no residual molten salt was detected in the ceramic layer. Therefore, the ceramic layer effectively provided a physical barrier during hot corrosion. The second reason is the thermal barrier effect of the ceramic layer. Generally, due to the poor thermal conductivity of YSZ, a temperature gradient forms within the TBC system, reducing the bond coating temperature by 50–170 °C depending on the ceramic layer thickness, which in turn decreases the oxidation rate of the bond coating.

In summary, the TBC (NiCrAlY/YSZ) exhibited the best resistance to Na_2_SO_4_ and mixed salt-induced hot corrosion [45,46].

## 5. Conclusions

In an air atmosphere at 900 °C, a composite salt film consisting of pure Na_2_SO_4_ and 25 wt.% NaCl leads to rapid and catastrophic thermal corrosion, forming non-protective corrosion products such as NiO and (Ni, Co)Cr_2_O_4_ spinel phases, which are prone to cracking and spalling. Under the pure Na_2_SO_4_ salt film, corrosion follows an alkaline melting mechanism, while under the NaCl-containing composite salt film, it is dominated by an active oxidation mechanism; following the application of NiCrAlY coatings and NiCrAlY/YSZ coatings to the M247 alloy surface via atmospheric plasma spraying (APS) technology, a continuous, dense, and highly stable α-Al_2_O_3_ oxide film forms on the NiCrAlY coating surface during thermal corrosion. This oxide film effectively resists both alkaline melting and active oxidation processes. The exceptional protective performance of the NiCrAlY/YSZ thermal barrier coating also stems from the dual effects of the physical and thermal barrier provided by the outer YSZ ceramic layer, ultimately endowing both coatings with outstanding thermal corrosion resistance. Subsequent research will simulate real-world gas turbine multi-medium coupled corrosion scenarios, supplement mechanical property testing such as coating thermal cycling stability, and conduct long-term service behavior and life prediction studies. These efforts will further expand the coating’s application potential in harsh environments, providing more comprehensive technical support for engineering practice.

## Data Availability

The original contributions presented in this study are included in the article. Further inquiries can be directed to the corresponding author.
